# A green approach for metoclopramide quantification in pharmaceutical products: new HPLC and spectrophotometric methods

**DOI:** 10.1038/s41598-024-59149-6

**Published:** 2024-04-16

**Authors:** İbrahim Demir, İbrahim Bulduk, Ibrahim A. Darwısh, Hüseyin Enginar

**Affiliations:** 1 Department of Chemistry, Faculty of Science & Arts, Afyon Kocatepe University, Afyonkarahisar, Turkey; 2 Department of Chemical Engineering, Faculty of Engineering, Afyon Kocatepe University, Afyonkarahisar, Turkey; 3 Department of Pharmaceutical Chemistry, College of Pharmacy, King Saud University, P.O. Box 2457, 11451 Riyadh, Saudi Arabia

**Keywords:** Metoclopramide, Pharmaceuticals, Green HPLC, Green spectrophotometry, AGREE, Environmental chemistry, Environmental impact, Environmental sciences

## Abstract

Green spectrophotometric and HPLC methods have been developed for the quantification of metoclopramide. In the spectrophotometric method, it was determined by direct absorbance measurement at 273 nm wavelength using ultrapure water as solvent. The Extend C18 column was used for the HPLC method. The mobile phase system consisted of a combination of ethanol and formic acid solution (pH 2.0; 30:70 v/v). Isocratic elution was applied and the flow rate was set at 1.0 mL min^−1^. Metoclopramide was detected at 273 nm. The methods performed were economical, rapid, environmentally friendly, and simple, providing metoclopramide analysis within 5 min. The methods have been successfully applied in pharmaceutical products without matrix interference. The results of the application of the developed methods to pharmaceutical products were statistically compared and no significant difference was observed between the methods. In addition, the greenness assessment of the developed methods was performed using AGREE software. Our developed methods, based on the use of solvents such as ethanol and water, are proposed as a more environmentally and analyst-friendly option for the quantification of metoclopramide in pharmaceutical products than other methods currently in use.

## Introduction

In recent years, the development of green analytical methods has attracted intense interest from researchers to reduce environmental impact and protect the health of analysts. High-performance liquid chromatography (HPLC) is widely used for drug analysis in the production and quality validation of pharmaceutical formulations. HPLC methods generally use a hydrophobic stationary phase and a polar mobile phase for effective separation^1,2^. C18, C8 and other column types used to separate different components in the sample are the basic units representing the stationary phase of HPLC. Columns provide the ability to speed up HPLC studies, a fast analysis process, and ultimately lower costs. A good column selection ensures acceptable chromatographic peaks and good separation for the analytes^3^. The mobile phases are usually a combination of water (with additives or buffer solutions to adjust the pH) and organic solvents such as acetonitrile/methanol. Acetonitrile and methanol are the most common organic solvents used in HPLC due to their full miscibility with water, low viscosity, and low chemical reactivity with device components and column surfaces^4,5^. During the development and validation of chromatographic methods, the focus is on analysis time as well as parameters such as accuracy, precision, and robustness. However, aspects of the environmental impact of the chromatographic method and the health of the analyst are still not adequately considered.

Unfortunately, methanol and acetonitrile have adverse effects on the environment and analyst health. Since a large amount of organic solvent wastes will be generated in HPLC analysis, it is now imperative to develop environment- and operator-friendly analytical methods and to replace polluting analytical methods with cleaner ones^6,7^. Whether organic solvents are environmentally friendly or not is evaluated according to health, safety, and environmental criteria and life cycle assessment^7^. Ethanol is one of the least harmful organic solvents to the environment and is used as an alternative solvent for environmentally friendly HPLC^8^. Ethanol is less hazardous than acetonitrile and methanol. Since it has a lower vapor pressure, it causes less evaporation and therefore less breathing. Additionally, ethanol has less of an environmental cost to dispose of than acetonitrile and methanol^9,10^.

Metoclopramide is a potent dopamine receptor antagonist used for its antiemetic properties. It is used in reduced gastrointestinal motility disorders, gastroesophageal reflux, and dyspepsia, and in nausea and vomiting associated with various gastrointestinal disorders, migraine, postoperative, and cancer treatment^11^. It is used in the form of hydrochloride salt in pharmaceutical formulations, because of its better bioavailability. Its physicochemical properties are presented in Table 1^12^.

**Table 1:** The physicochemical properties of Metoclopramide.

Properties		Value
CAS No:		364-62-5
Structure		
Molecular weight		299.80
Melting point		171–173 °C
Log P		2.667
pKa	Acidic	14.49
	Basic	9.04
Solubility		It is soluble in water and organic solvents such as ethanol

Analytical methods previously published for the quantification of metoclopramide in bulk^13–15^, pharmaceutical dosage forms^16–18^, and biological fluids were scanned in the literatüre^19–21^. Spectrophotometric^22–24^, high-performance liquid chromatographic^25–27^, and liquid chromatographic coupled with tandem mass spectrometry (LC–MS/MS)^28^ methods were reported for determination of metoclopramide in pharmaceutical dosage forms and biological fluids were developed.

Many of these methods necessitate the use of harmful and specific chemicals. During the literature search, no green HPLC and spectrophotometric technique for the quantification of metoclopramide in pharmaceutical formulations was found. Therefore, this study aimed to develop and validate a spectrophotometric method using ultra-pure water as a solvent and a liquid chromatography method using environmentally and operatör friendly ethanol for the quantification of metoclopramide in pharmaceutical products by a simple extraction procedure. Ethanol is seen as a more environmentally friendly substitute for methanol and acetonitrile. This study shows that standard mobile phases provide a highly satisfactory performance when replaced with less hazardous chemicals and “more environmentally friendly” solvents.

## Experimental

### Instrument

A Shimadzu UV 1800 Double-Beam UV–VIS spectrophotometer (Kyoto, Japan) was used for spectrophotometric analysis. The system uses UV Probe 2.52 software. Experiments were carried out at the UV region (200.0–400.0 nm) using quartz cuvettes.

An Agilent 1260 series (Agilent Technologies, USA) LC system was used for HPLC analysis.

The compounds were separated using the Extend C18 (5.0 μm; 4.6 × 150.0 mm) column (Agilent, USA).

### Materials

For liquid chromatography, all solvents were of gradient purity. European Pharmacopoeia (EP) Reference Standard (Metoclopramide Hydrochloride), analytical grade formic acid (≥ 99.0%), and ethanol (≥ 99%) were bought from Sigma-Aldrich (Istanbul, Turkey). Metoclopramide tablets (METPAMID, 10 mg) were obtained from a local pharmacy for use in this investigation. All solutions and the mobile phase were prepared with ultrapure water (0.055 µS cm^−1^). The mobile phase was filtered by passing through a membrane filter (0.45 µm) in a vacuum pump and sonicated before analysis.

### Preparation of standard solutions

The active pharmaceutical ingredient (Metoclopramide HCl) equivalent to 25 mg of Metoclopramide was transferred to a 50 mL volumetric flask containing 30 mL of ultrapure water. It was sonicated in an ultrasonic bath until a clear solution was obtained and the volume was made up to 50 mL with ultrapure water. Thus, the stock standard solution was prepared. Aliquots of 0.1, 0.2, 0.3, 0.4, 0.5, 0.5, and 1.0 mL of the stock standard solution were transferred into 10 mL volumetric flasks and diluted with ultrapure water to a final concentration of 530 μg mL^−1^.

### Preparation of sample solution

For the quantitative analysis of metoclopramide in commercial formulations, ten (METPAMIDE, 10 mg) tablets were precisely weighed and the mass of an average tablet was recorded. The tablets were crushed in a clean mortar, powdered until very fine and mixed homogeneously. The tablet powder containing 25 mg of metoclopramide was precisely weighed and dissolved in 50 mL of ultra-pure water, and to complete extraction of the drug, it was subjected to sonication for 30 min. The solution was allowed to precipitate suspended insoluble matter for approximately 1 h and filtered through a 0.45 μm membrane filter to remove the insoluble substances. This solution was called the tablet sample solution. The sample solution was prepared by taking 500 µL from the stock solution and diluting it to 10 mL with ultra-pure water. It was quantitatively analyzed by chromatographic and spectrophotometric methods.

### Determination of the wavelength

Spectrophotometric studies were carried out using a Shimadzu UV 1800 double beam spectrophotometer (Shimadzu, Japan) and UV-Probe software. Absorbances of solutions were determined using a 1.00 cm quartz cell against a blank sample. The working standard solutions of metoclopramide at six concentrations from 5 to 30 µg mL^−1^ were scanned on a spectrophotometer at a wavelength range of 200–400 nm.

### Development of methods

For spectrophotometric analysis, the spectral patterns of metoclopramide in different solvents were studied in detail. Ultrapure water was used as the solvent for spectrophotometric analyses since the best spectra were obtained when ultrapure water was used as the solvent. In addition, since the maximum absorbance values of the standard solutions were obtained at 273 nm wavelength, the absorbance values of the standard and sample solutions were measured at this wavelength. Chromatographic conditions were optimized using different mobile phase combinations, different column types/sizes, and different temperatures to get good peak parameters such as a good peak shape, lowest tailing factor, a short retention time, and a high theoretical plate number.

### Valıdation procedure of methods

HPLC and spectrophotometric methods developed for the quantification of metoclopramide in pharmaceutical preparations have been validated according to International Conference on Harmonisation (ICH) Q2 (R1) guidelines for selectivity, system suitability, linearity, precision, sensitivity, and robustness^29,30^.

#### Linearity

The linearity of the HPLC method was determined by injecting 10 µL of six standard solutions with a concentration range of 5–30 µg mL^−1^ into the HPLC system in triplicate on three different days. The peak areas obtained for each concentration were recorded. Calibration graphs were created with concentrations on the x-axis and peak areas on the y-axis. The linearity of the spectrophotometric method was determined by measuring the absorbance values of six standard solutions in the concentration range of 5–30 µg mL^−1^ in triplicate on three different days in the spectrophotometer. The absorbance values obtained for each concentration were recorded. Calibration graphs were created with concentrations on the x-axis and spectrophotometric responses (absorbance values) on the y-axis. Regression analysis was performed using the least squares method with the data obtained from both analytical methods.

The linearity of the methods was measured by the slope, intercept, correlation coefficient, and absolute mean recovery value of the regression equations.

#### Accuracy

The accuracy of the methods was evaluated by determining the % recovery values of the added analyte using the “standard addition method”. For this, three different amounts of powder standards were added to three separate sample solutions (25 µg mL^−1^) at the rate of 50%, 100%, and 150% of the analyte content and mixed. The obtained solutions were analyzed by the developed methods and their analyte contents were determined. The recovery values of the added standard amount were computed. Triplicate analyses were performed for each concentration level. The percentage recovery values of the standard amount added have been calculated. Three copies of the analysis were performed for each concentration level.

#### Precision

The precision of the methods was evaluated as intraday/interday precision. Intraday precision was determined by quantitative analysis of the standard solution (25 μg mL^−1^) on the same day (n = 3). Inter-day precision was determined by quantitative analysis of the standard solution (25 µg mL^−1^) on three consecutive days (n = 9). In the HPLC method, the peak areas and retention times were recorded and the relative standard deviation values were calculated. In the spectrophotometric method, absorbance values were measured and relative standard deviation values were calculated.

#### Robustness

To assess the robustness of the chromatographic method, small deliberate changes were made to the method conditions, such as the organic solvent content in the mobile phase (± 2%), the flow rate of the mobile phase (± 0.1 mL min^−1^), and the detection wavelength (± 2 nm). After each modification, the standard solution (25 μg mL^−1^) was injected into the chromatographic system and the results were compared with the results under the original chromatographic conditions. System suitability parameters were recorded for each case. The effects of the changes were investigated by triplicate analysis of the standard solution. Minor changes in detection wavelength (271 and 275 nm) and in organic solvents (ethanol and isopropyl alcohol) were made to evaluate the robustness of the spectrophotometric method. The results were compared with those under the original spectrophotometric conditions. The effects of the changes were investigated by triplicate analysis of the standard solution.

#### Limit of Detection (LOD) and Limit of Quantification (LOQ)

The standard deviation technique was used to determine the limit of detection (LOD) and limit of quantification (LOQ) of the proposed methods. For this, blank samples (samples without metoclopramide) were prepared. For the HPLC method, the blank solution was injected into the system in triplicate and the chromatographic responses (peak areas) of these blank samples were recorded. For the spectrophotometric method, the blank solution was analyzed by the spectrophotometric method in triplicate and the spectrophotometric responses (absorbance values) were recorded. LOD and LOQ values were calculated from the formulas LOD = 3.3 × SD/S and LOQ = 10 × SD/S using the slope (S) of the calibration curve and the standard deviation (SD) of the peak area.

#### Specificity

The specificity of the methods was evaluated by determining whether the analyte was completely separated in the presence of other excipients normally found in dosage forms and by examining the presence of interfering impurities. To evaluate the specificity of the chromatographic method, mobile phase, standard, and sample solutions were injected into the HPLC system. The chromatogram of the sample solution was compared with the chromatogram of the standard solution. The presence of interfering peaks during the retention time of the analyte peak was examined. To evaluate the specificity of the spectrophotometric method, spectra of the standard, sample and solvent (ultrapure water) were scanned in a spectrophotometer in the wavelength range of 200–400 nm. The obtained spectra were compared and the presence of interfering bands was examined.

#### System suitability

To evaluate the system suitability of the HPLC method, the standard solution (25 µg mL^−1^) was injected into the HPLC system six times at short regular intervals. Retention times, peak areas, theoretical plate numbers, and tailing factors of metoclopramide peaks were recorded. Relative standard deviation values of retention times and peak areas were calculated. To evaluate the system suitability of the spectrophotometric method, the absorbance values of the standard solution (25 µg mL^−1^) were measured at short regular intervals. Relative standard deviation values of absorbance values were calculated.

#### Application of methods to commercial formulations

For the quantitative analysis of metoclopramide in commercial formulations, ten (Metpamide, 10 mg) tablets were precisely weighed and the mass of an average tablet was recorded. The tablets were crushed in a clean mortar, ground into a fine powder and mixed homogeneously. The tablet powder was precisely weighed and dissolved in 50 mL of ultra-pure water, and to complete extraction of the drug, it was subjected to sonication for 30 min. The injector tip was filtered by passing through the filter to remove the insoluble substances. This solution was called the sample stock solution. The sample solution was prepared by taking 500 µL from the stock solution and diluting it to 10 mL with ultra-pure water and analyzed quantitatively using the developed methods.

#### Assessment of greenness profiles

AGREE Analytical Greennes metric software (the version of v. 0.5 beta, available at https://mostwiedzy.pl/wojciech-wojnowski,174235-1/AGREE) was used to assess the greenness of the developed approaches. AGREE is a metric system that uses important principles to assess the greenness of analytical techniques. The greenness score is a weighted average of the benchmark values and is displayed in the chart’s center, rounded to two decimal places. The greenness score goes from 0.00 to 1.00. A score of less than 0.50 indicates that the method is undesirable, a score of 0.50–0.75 suggests that it is acceptable, and a score of greater than 0.75 indicates that it is good^31–33^.

## Results and discussion

### Determination of detection wavelength

**Figure 1:**
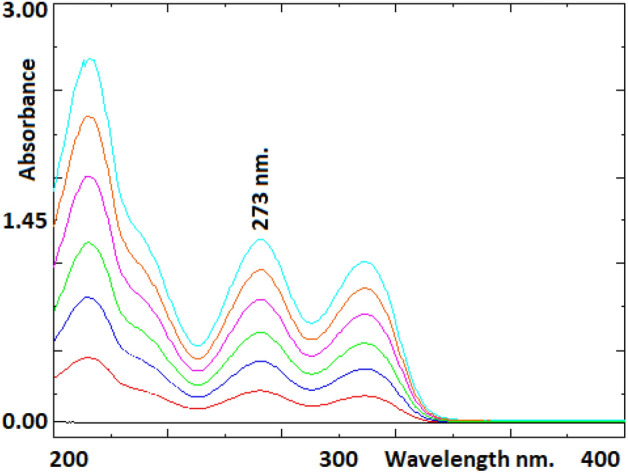
The overlay spectrum of metoclopramide standard solutions in the concentration range of 5–30 µg mL^−1^.

Standard solutions prepared with ultra-pure water were scanned in the range of 200–400 nm wavelength range on a spectrophotometer. Metoclopramide had maximum absorption at a wavelength of 273 nm. The overlay spectrum of metoclopramide standard solutions is presented in Fig. 1.

### Method conditions

#### HPLC method conditions

An Extend C_18_ column (150 × 4.6 mm, 5 μm) was used for separation and the column temperature was kept constant at 25 °C. The combination of ethanol and formic acid solution (pH 2.0; 30:70 v/v) was used as the mobile phase and isocratic elution was performed at a flow rate of 1.0 mL min^−1^. 10 µL of standard and sample solutions were injected into the system. Metoclopramide was detected at a wavelength of 273 nm.

#### Spectrophotometric method conditions

Ultrapure water was used as a solvent for spectrophotometric analysis because the best spectra were obtained when ultrapure water was used as a solvent. The absorbance values of metoclopramide solutions in ultrapure water were measured directly in the spectrophotometer. Furthermore, since the maximum absorbance values of the standard solutions were obtained at a wavelength of 273 nm, the absorbance values of the standard and sample solutions against ultrapure water were measured at this wavelength.

### Valıdation results of methods

#### Specificity

**Figure 2:**
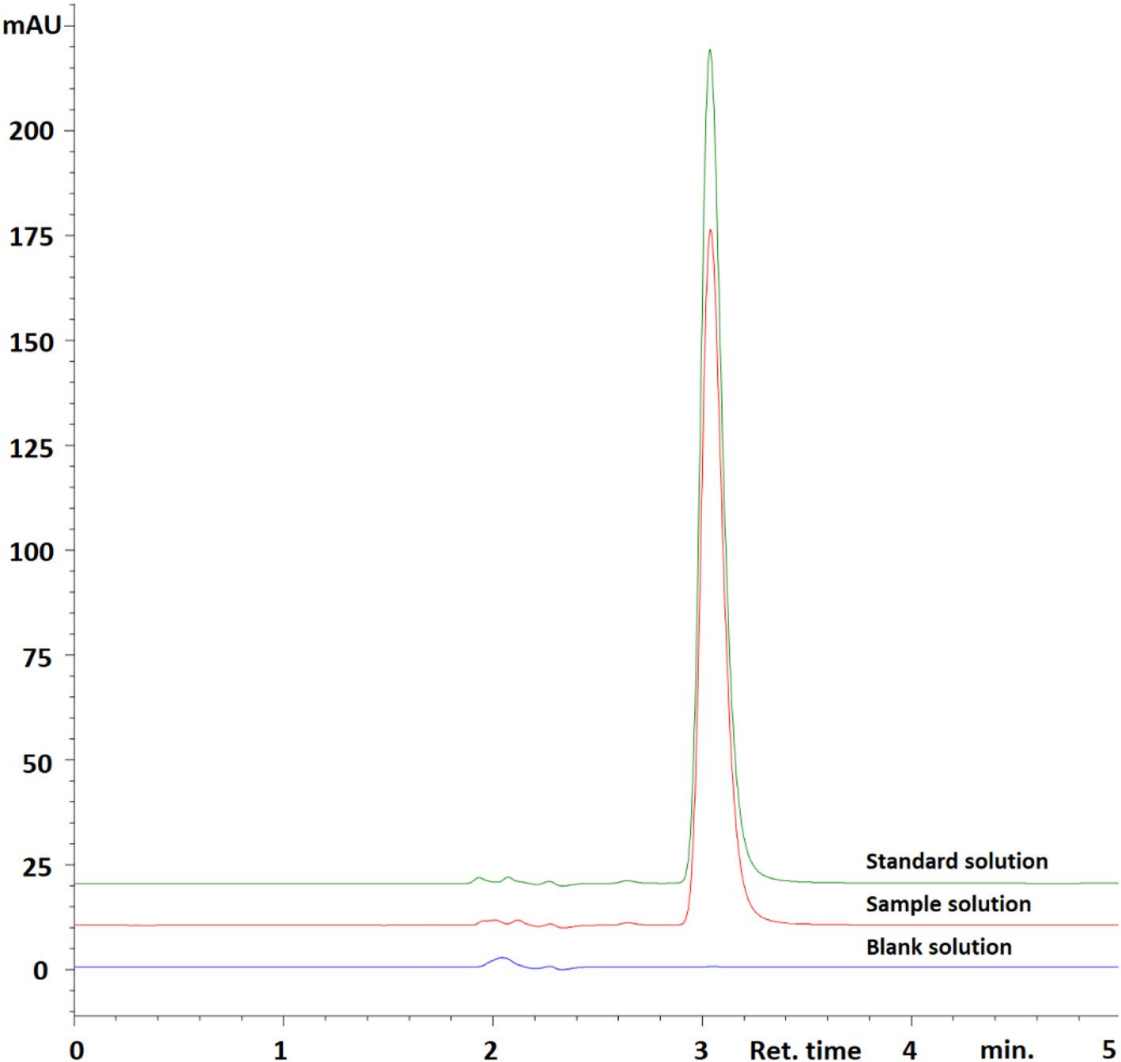
Chromatograms of mobile phase as the blank, sample, and standard solutions obtained with the HPLC method.

In order to evaluate the specificity of the HPLC method, chromatograms of the sample, mobile phase, and standard solutions were compared. No interfering peak(s) were observed during the retention period of the analyte peak. Chromatograms of mobile phase as the blank, sample, and standard solution obtained with the HPLC method are presented in Fig. 2.

**Figure 3:**
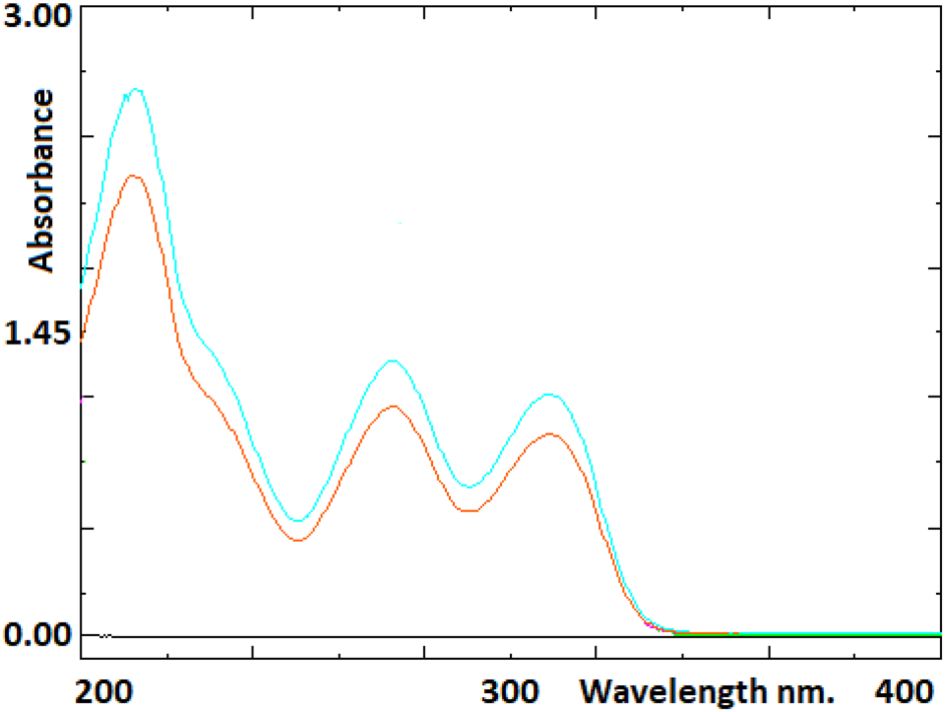
Spectrums of mobile phase as the blank, sample, and standard solution obtained with the spectrophotometric method.

Spectrums of ultra-pure water used as standard, sample and solvent were taken to evaluate the specificity of the spectrophotometric method. The three spectra were compared and analyzed in terms of the spectral band(s) causing interference around the analyte spectrum. No bands interfering with metoclopramide bands were observed in all spectra. Similarly, spectrums of mobile phase as the blank, sample, and standard solution obtained with the spectrophotometric method are presented in Fig. 3.

#### System suitability test

The primary parameters for the system conformity tests have been determined and are listed in Table 2. It was observed that metoclopramide has excellent peak symmetry. In addition, consistently low variability was observed in the retention times and peak areas of metoclopramide. The correlation coefficients of the calibration curves are greater than 0.999, which shows that the methods are suitable even for samples with complex matrices. The results of the system conformity tests show that the developed methods are suitable for metoclopramide quantification.

**Table 2:** The results of the system suitability tests.

Sample	HPLC method				Spectrofotometric method
	Peak area	Retention time Min.	Peak tailing	Theoretical plate number	Absorbance
1	1248.88	3.038	1.385	3177	1.081
2	1257.60	3.036	1.371	3142	1.066
3	1248.52	3.038	1.378	3167	1.067
4	1249.70	3.037	1.366	3111	1.080
5	1253.15	3.038	1.370	3143	1.068
6	1258.10	3.035	1.384	3155	1.083
Average	**1252.66**	**3.037**	**1.376**	**3149**	**1.074**
S.D.	**4.346**	**0.001**	**0.008**	**23.121**	**0.008**
R.S.D %	**0.347**	**0.042**	**0.576**	**0.734**	**0.739**

Significant values are in [bold].

#### Linearity

For the HPLC method, standard solutions were injected into the system and the peak areas and retention times of metoclopramide were recorded. The average peak area and retention time were calculated for each concentration level. A calibration graph was created with the peak area against the standard solution concentration. For the spectrophotometric method, the absorbance values of standard solutions were measured against the empty solution. The average absorbance value was calculated for each concentration level. A calibration graph was created with absorbance values against standard solution concentrations.

The linearity data of the analytical methods were evaluated by regression analysis. Linear regression analysis based on the least squares approximation was used to determine the slope and intersection of the regression equation. The results of the linearity studies are presented in Table 3. The calibration curve showed a good linear relationship in the concentration range of 5–30 µg mL^−1^.

**Table 3:** Linearity data of analytical methods.

Parameter	HPLC method	Spectrophotometric method
Range of concentration [μg mL^−1^] [*n* = 6]	5–30	
The slope of the regression equation	50.0930	0.0435
The intercept of the regression equation	2.8095	− 0.0083
Correlation coefficient	0.9997	0.9995
Limit of detection [μg mL^−1^]	0.70	0.90
Limit of quantification [μg mL^−1^]	2.00	2.80
Recovery % [*n* = 3]	99.52–100.58	98.99–101.07

#### Precision

The results of intraday and interday sensitivity tests are summarized in Table 4. As a result of both tests, it was determined that the relative standard deviation values were less than 1.0%. These results demonstrate reasonable reproducibility for the developed methods.

**Table 4:** Results of the precision of the methods.

Precision		HPLC method			Spectrophotometric method	
		Retention time, min.	Peak area	Assay %	Absorbance	Assay %
Intraday	Average	3.038	1253.68	100.00	1.070	100.00
	SD	0.001	0.781	0.062	0.001	0.108
	RSD %	0.033	0.062	0.062	0.108	0.108
Interday	Average	3.037	1252.67	100.00	1.074	100.01
	SD	0.002	1.527	0.122	0.002	0.135
	RSD %	0.079	0.122	0.122	0.135	0.135

#### Accuracy

For the accuracy of the methods, the solutions prepared by spiking the metoclopramide standard at the rate of 75%, 100%, and 125% of the metoclopramide content to the sample solution (25 µg mL^−1^) were analyzed by the developed methods. The % recovery values of the standard amounts added to the sample solution were calculated. The recovery percentages ranged between 99.49 and 99.86% for the HPLC method and between 99.31 and 99.74% for the spectrophotometric method. The maximum relative standard deviation values are 0.282 for the chromatographic method and 0.476 for the spectrophotometric method. The results of the recovery studies are presented in Table 5.

**Table 5:** Accuracy data of chromatographic methods.

Method	Std. addition level %	Std. addition amount μg mL^−1^	Mean recovery %	S. D.	R. S. D. %
HPLC method	75	18.75	99.61	0.304	0.305
	100	25.00	99.84	0.224	0.224
	125	31.25	99.98	0.156	0.156
Spectrophotometric method	75	18.75	99.33	0.498	0.501
	100	25.00	99.69	0.295	0.296
	125	31.25	99.84	0.267	0.267

#### Robustness

To test the robustness of the developed methods, small deviations from standard values were made in the method parameters, and the effects of these deviations on the results were investigated. This study was carried out at a concentration level of 25 µg mL^−1^. According to the results obtained, the highest relative standard deviation value was calculated as 0.410%. The results of the robustness tests are given collectively in Table 6.

**Table 6:** Results of robustness tests of analytical methods (n = 3).

Method	Conditions	Values	Average recovery %	R.S.D. %
HPLC method	Standard method conditions		99.99	0.327
	The high flow rate of the mobile phase	1.10 mL min^−1^	99.67	0.397
	The low flow rate of the mobile phase	0.90 mL min^−1^	99.52	0.410
	The high-detection wavelength	275 nm	99.70	0.231
	The low-detection wavelength	271 nm	99.75	0.332
	The high amount of ethanol in the mobile phase	32%	99.72	0.264
	The low amount of ethanol in the mobile phase	28%	99.60	0.309
Spectrophotometric method	Standard method conditions		99.93	0.394
	The high-detection wavelength	275 nm	99.53	0.341
	The low-detection wavelength	271 nm	99.34	0.453
	Solvent	Ethanol	99.45	0.407
	Solvent	Isopropyl alcohol	99.17	0.400

### Quantitative analysis of metoclopramide in marketed tablets

6 tablets containing 10 mg of metoclopramide each were analyzed quantitatively using the developed methods. Quantitative analysis results, standard deviation and relative standard deviation values are presented in Table 7. The results obtained with the developed methods were compared in terms of averages by Student (T) test and in terms of standard deviations by Fischer (F) test. When the results are examined, it is seen that In terms of accuracy and precision, the methodologies do not differ much from one another. T and F values computed are lower than those stated in the relevant tables.

**Table 7:** Quantitative analysis results of marketed tablets.

Sample	HPLC method		Spectrophotometric method	
	mg/tablet	%	mg/tablet	%
1	10.16	101.53	9.99	99.97
2	9.93	99.23	9.93	99.37
3	10.13	101.23	10.02	100.27
4	9.98	99.73	9.97	99.77
5	10.00	99.93	9.93	99.37
6	9.84	98.33	10.12	101.27
Average	10.01	100.00	9.99	100.00
S.D.	0.11	1.21	0.07	0.71
R.S.D.	1.10	1.21	0.71	0.71
t_value_/t_table_	0.2080/2.5706			
F_value_/F_table_	2.8868/5.0503			

### Assessment of greenness profiles

**Figure 4:**
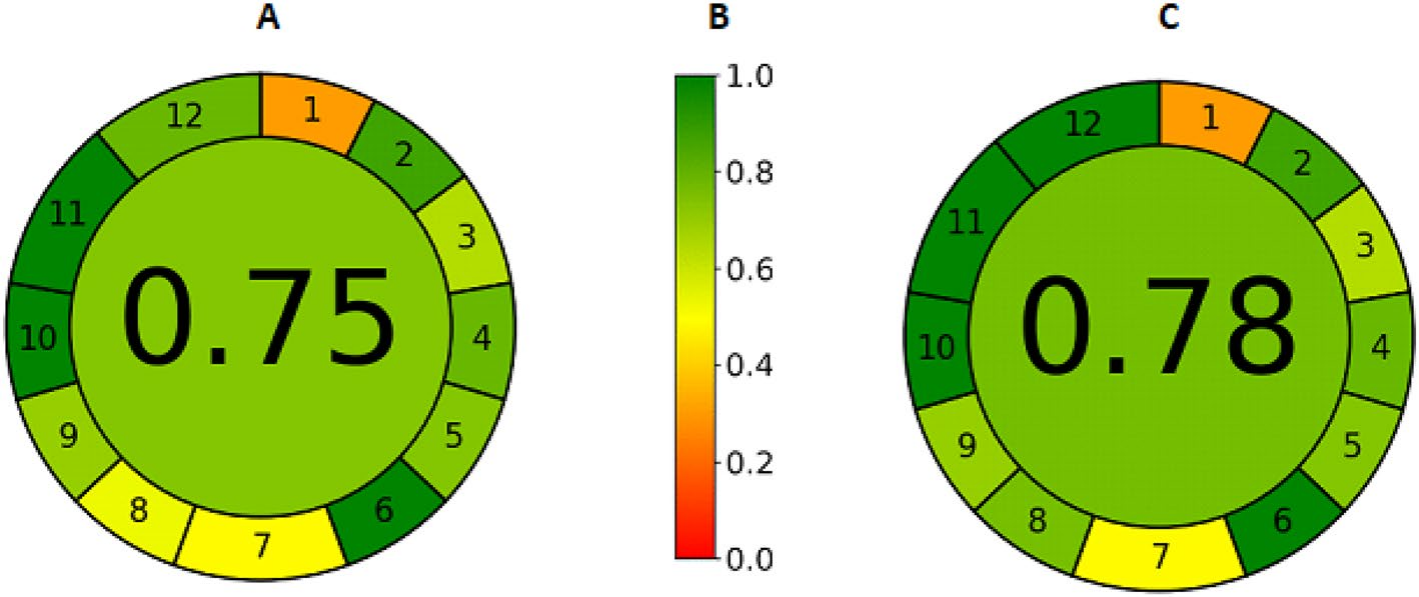
(**A**) AGREE pictograms of HPLC method. (**B**) AGREE Pictogram scale. (**C**) AGREE pictograms of UV-Spectrophotometric method.

The AGREE pictograms of the developed methods are presented in Fig. 4. The AGREE analytical scores of the developed HPLC and spectrophotometric methods were 0.70 and 0.75, respectively. In the AGREE greenness assessment, a score below 0.50 indicates that the method is unacceptable, a score between 0.50 and 0.75 is acceptable and a score above 0.75 indicates that the method is excellent. Based on the AGREE analytical scores obtained in this study, it can be concluded that both analytical methods proposed for the quantification of metoclopramide are excellent green techniques.

The AGREE tool proves that the Green Chemistry Principles have been achieved. However, the energy consumption parameter is an important point when using chromatographic and spectrophotometric techniques because the environmental impact produced can be analyzed and measured by the so-called carbon footprint. Carbon footprint is a metric factor expressed in kg CO_2_ equivalent for assessing the negative impact of a methodology that includes the device's power, operating time and electricity emission factor. This method is calculated with the expression provided in ESI S1 within the HEXAGON tool described by Ballester-Caudet et al., in 2019^34^.

The total carbon footprint for the HPLC method was 0.00765 kg CO_2_ equivalent, while for the spectrophotometric method it was 0.00550 kg CO_2_ equivalent. According to the HEXAGON approach, the final score is 0 out of five points because the total calculated carbon footprint is less than 0.1.

Since the total carbon footprint is less than 0.1, the overall competency score is 0 on a 5-point scale. The shorter the analysis time (only 5 min in our methods), the lower the carbon footprint score and the more environmentally friendly the method. Additionally, when using the HPLC method, a significant reduction in total cost is achieved, as the amount of solvent required when performing multiple analysis is noticeably reduced compared to single-component analysis. Many factors are taken into account regarding the cost sustainability of the method; for example, overall analysis time, number of samples performed per seven days, and equipment cost. Performing 50 or more samples per week is considered the most environmentally friendly approach. In our example the number of samples analyzed in seven days is approximately 500, which means a high green value in terms of economic cost according to the HEXAGON tool description^35,36^.

### Discussion

The chromatographic and spectrophotometric behavior of metoclopramide was investigated in this work utilizing solvents that were both ecologically and operator-friendly. The environmental friendliness of the developed methods was evaluated from the sample preparation step to the detection step. Only water was used in the sample preparation stage and it was quite simple. While ethanol, which is safe for the environment and analyst health, was used as an organic modifier in chromatographic analyses, ultrapure water was used as a solvent for spectrophotometric analyses. The developed methods were validated according to ICH guidelines and their method performance was found to be excellent. Metoclopramide was selectively analyzed by our methods developed with high linearity, precision, accuracy, specificity, and robustness. The detection and quantification limits of the developed methods are very low. Additionally, system suitability parameters showed that the performance of the methods was sufficient. One of the important results of this study is that the waste is non-toxic. All requirements of the validation process have been met without compromising the quality of performance and the study has achieved its purpose. This study has shown that ethanol and water-based mobile phases can be successfully applied in pharmaceutical analyses and that spectrophotometric analyses can be performed using only water. A comparison of the new HPLC method with the old ones is presented in Table 8.

**Table 8:** Comparison of the new method with the old ones.

Ref. no.	Mobile phase	Stationary phase	Linearity	LOD/LOQ	Aplication	Disadvantage
^25^	Acetonitrile, Methanol and Water with a ratio of 25:25:50	ZORBAX SB-C18 (4.6 × 250 mm, 5.0 µm) column	2–20 µg/mL R2 = 0.999	0.052/0.159 µg/mL	Tablet formulation	Acetonitrile Methanol
^26^	Acetonitrile (18%) in 0.02 M ammonium acetate containing 0.1% triethylamine	Novapak C18 4 pm (3.9 × 150 mm) column	0.025–5 µg/mL R2 = 0.997	–	Rat serum	Acetonitrile
^27^	Acetonitrile, 20 m M Potassium dihydrogen phosphate buffer solution (pH 3 adjusted with orthophosphoric acid) in the ratio of 40:60	Waters C18 3.9 × 300 mm µBondapak	5–75 µg/mL R2 = 0.997	0.75/2.24 µg/mL	Tablet formulation	Acetonitrile
Proposed HPLC Method	Ethanol and formic acid solution (pH 2.0; 30:70 v/v)	Extend C18 (5.0 μm; 4.6 × 150.0 mm) column	5–30 µg/mL R2 = 0.9997	0.70/2.00 µg/mL	Tablet formulation	No Acetonitrile No methanol

## Conclusions

Clean water resources on earth are rapidly decreasing and air pollution poses a major problem for the future of humanity. In this regard, the development of environmentally friendly methods for preventing environmental pollution, waste management, and reducing energy consumption is much more important for the future of humanity. The proposed method is suggested as a more environmentally friendly option than other techniques currently in use for the determination of the amount of metoclopramide in pharmaceutical products. The developed methods offer an environmentally and operator-friendly approach based on the use of highly environmentally friendly solvents such as ethanol and water for the analysis of metoclopramide in pharmaceutical products. The proposed methods have been successfully applied to determine metoclopramide in commercial dosage forms. The AGREE assessment tool was used to evaluate the degree of greenness and it was confirmed that the proposed method is a green method. In the literature review, no method using a mobile phase containing less toxic solvents has been found for the determination of metoclopramide. In conclusion, the findings of this study could be a precursor to the transformation of conventional chromatographic methods into more environmentally friendly methods while maintaining satisfactory performance values of this method.

## Data Availability

The datasets used and/or analysed during the current study available from the corresponding author on reasonable request.
